# Pleural effusion in a child with a ventriculoperitoneal shunt and congenital heart disease

**DOI:** 10.1186/s40064-016-1738-z

**Published:** 2016-01-27

**Authors:** Jennifer Henningfeld, Rohit S. Loomba, Santiago Encalada, Kristin Magner, Jennifer Pfister, Anne Matthews, Andrew Foy, Theresa Mikhailov

**Affiliations:** Department of Pulmonary and Sleep Medicine, Children’s Hospital of Wisconsin/Medical College of Wisconsin, 9000 W. Wisconsin Ave MS B620, Milwaukee, WI 53226 USA; Department of Cardiology, Children’s Hospital of Wisconsin/Medical College of Wisconsin, 9000 W. Wisconsin Ave, MS 713, Milwaukee, WI 53226 USA; Department of Critical Care, Children’s Hospital of Wisconsin/Medical College of Wisconsin, 9000 W. Wisconsin Ave MS 681, Milwaukee, WI 53226 USA; Department of Neurosurgery, Children’s Hospital of Wisconsin/Medical College of Wisconsin, 999 N 92nd St. Suite 310, Milwaukee, WI 53226 USA

**Keywords:** Effusion, Shunt, Ventriculoperitoneal, Ventriculoatrial, Congenital heart disease

## Abstract

We present the unique case of an 8 month old infant who required extracorporeal membrane oxygenation (ECMO) after neonatal repair of tetralogy of Fallot. While on ECMO, he developed grade 3 intraventricular hemorrhage resulting in hydrocephalus requiring ventriculoperitoneal (VP) shunt placement at 5 months of life. He presented to cardiology clinic with a 2-month history of poor weight gain, tachypnea, and grunting and was found to have a large right sided pleural effusion. This was proven to be cerebrospinal fluid (CSF) accumulation secondary to poor peritoneal absorption with subsequent extravasation of CSF into the thoracic cavity via a diaphragmatic defect. After diaphragm repair, worsening ascites from peritoneal malabsorption led to shunt externalization and ultimate conversion to a ventriculoatrial (VA) shunt. This is the second reported case of VA shunt placement in a child with congenital heart disease and highlights the need to consider CSF extravasation as the cause of pleural effusions in children with VP shunts.

## Case report

We present the case of an 8 months old diagnosed postnatally with tetralogy of Fallot (TOF). Permission to report the following medical and personal information was obtained from the parents of the patient.

He underwent neonatal repair due to early cyanotic spells that were not oxygen responsive. Subsequent to his cardiac repair, he required extracorporeal membrane oxygenation (ECMO) support for right ventricular dysfunction and permanent pacemaker placement for atrioventricular block. While on ECMO, the infant developed grade 3 intraventricular hemorrhage (IVH) and resulting hydrocephalus, requiring placement of a ventriculoperitoneal shunt (VP) shunt at 5 months old. Due to the degree of systemic right ventricular dysfunction and the immediate need for elevated cardiac support, creating an atrial septal defect was not considered to be a reasonable approach.

At 8 months old, he presented to cardiology clinic with mild respiratory distress reportedly starting 2 months prior. He also had suboptimal weight gain over the previous 2 months despite adequate caloric intake. On exam he was tachypneic and was saturating above 90 %. A complete metabolic panel was normal except for a bicarbonate level of 17.1. Brain natriuretic peptide was 137, down trending since his TOF repair. A complete blood count and erythrocyte sedimentation rate were normal; his initial capillary blood gas demonstrated carbon dioxide partial pressure of 33.1. A chest radiograph revealed a right sided pleural effusion with intact left-sided VP shunt catheter. The infant was admitted to the pediatric intensive care unit (PICU) for further evaluation and management of the effusion.

A right pleural chest tube was placed, resulting in marked improvement in the infant’s work of breathing. Continued daily output in excess of 400 cc raised concern for diaphragmatic defect with extravasation of fluid from the abdomen into the thorax.

Pleural fluid analysis demonstrated a transudative profile with elevated beta 2 transferrin levels consistent with cerebrospinal fluid (CSF). The decision was made for diaphragmatic exploration and drainage of the right pleural effusion. Exploration revealed a right sided diaphragmatic hernia with omentum and CSF in the right pleural cavity and the hernia was repaired.

Following diaphragm repair, the infant had abdominal distention, discomfort, and grunting. These symptoms raised concern for impaired peritoneal absorption of CSF producing increased abdominal pressure now that no diaphragmatic defect allowed for decompression. Abdominal ultrasound demonstrated diffuse fluid throughout the abdomen and pelvis. Shunt externalization and drainage of peritoneal fluid improved the distention and respiratory distress.

After monitoring central venous pressure (CVP) for several days, his right atrial pressure was determined amenable for ventriculoatrial (VA) shunt placement. CVPs ranged from 10 to 12 mmHg during this period. It was believed that these were elevated due to residual right ventricular dysfunction. The infant tolerated VA shunt placement and was discharged with no respiratory or abdominal symptoms. He continues to follow-up in cardiology and neurosurgery clinic without concern.

## Discussion

We present the case of an infant with TOF requiring neonatal repair and postoperative ECMO support. IVH while on ECMO led to hydrocephalus requiring VP shunt placement. This shunt had to be converted to a VA shunt due to poor peritoneal absorption of CSF, a phenomenon recognized by a persistent right sided pleural effusion secondary to a diaphragmatic hernia.

This is the second reported case of VA shunt placement in a child with congenital heart disease (CHD), the first in a patient with TOF. The first patient was a 15-year-old girl with a history of congenital pulmonary stenosis and aortic insufficiency. A VP shunt for hydrocephalus was placed at 2 years of age, but at age 15 she underwent colectomy for juvenile colonic polyposis. Postoperatively, ascites developed, requiring VP to VA shunt conversion. During reported follow-up she did well with no cardiac or shunt-related issues (Niggemann et al. 1990).

In the reported case, the decision to perform a complete neonatal repair was made based on our institutional practice to proceed with neonatal repair in infants without contraindications for cardiopulmonary bypass. This has become standard practice at our institution for patients requiring neonatal intervention as good outcomes have been achieved with this strategy without the risk of systemic to pulmonary shunt clot. At our institution, we have found no difference in perioperative factors such as bypass time, cross clamp time, and deep hypothermic circulatory arrest time for neonatal versus non-neonatal repair, with or without earlier palliation. In follow-up we have found only a mild increase in need for a trans-annular patch and postoperative arrhythmias. This increase in risk is lower than that of a systemic to pulmonary shunt clot while awaiting complete repair.

The question in CHD patients becomes whether the right heart will tolerate the additional volume load due to CSF drainage. If cardiac function is normal, it is unlikely that this volume should be an issue. In our case, the daily CSF output was approximately 300 cc after shunt externalization. In the setting of normal right ventricular function with a cardiac output of at least 2500 ml/min, this rate of drainage would increment his cardiac output to 2500.2 ml/min. A VA shunt is associated with an additional minor volume load when compared to a VP shunt or physiological resorption of CSF into the sagittal sinus since there is now a direct connection into the atrium. While CSF absorbed in the peritoneum may eventually return to the heart via the lymphatic system and the caval veins, the direct volume burden associated with this is felt to be lower than that of a shunt draining directly into the atrium.

The additional volume burden on the heart being negligible, the next issue becomes the risk of right heart failure, which can cause an increased CVP and, theoretically, reduce CSF flow down the shunt. If the CVP increased above that of the cerebral ventricle, this pressure differential would prevent adequate CSF drainage and lead to elevated intracranial pressure. Therefore, VA shunt placement in a patient with right-sided heart failure and increased CVP may be ineffective for controlling hydrocephalus. On the other hand, in surgically palliated CHD, CVPs often range from 5 to 12 mmHg with normal right ventricular function. As normal pressures throughout the intracranial ventricular system range from 10 to 20 mmHg, the CSF pressure should exceed the CVP in most patients with CHD (Gilland 1969).

While the hemodynamics in patients with CHD seems amenable to placement of a VA shunt, there are additional considerations regarding their long-term consequences. Specifically, there are case reports and case series describing development of pulmonary hypertension in patients with VA shunts. This is believed to be secondary to chronic thromboembolic events damaging the pulmonary vasculature (Kim and Lang 2012; Kluge et al. 2009, 2010; Vernet and Rilliet 2001; Pascual and Prakash 1993; Piatt and Hoffman 1989; Drucker et al. 1984; Fagan and Jennings 1978).

Pediatric data on this comes primarily from Piatt et al. who reported 4 cases of fatal cor pulmonale in children with VA shunts and otherwise normal hearts. These children died at a median age of 6 (range 2–7 years) with all demonstrating evidence of pulmonary emboli (Piatt and Hoffman 1989). Patients with right-sided CHD, such as our patient, may have a compounded risk of pulmonary hypertension and subsequent right ventricular dysfunction. Nevertheless, this is merely speculation as this is only the second reported case of a VA shunt in a child with CHD (Niggemann et al. 1990).

This case also highlights the need to consider CSF extravasation as the cause of pleural effusions in patients with VP shunts. Other conditions more classically associated with such effusions must also be addressed, including congestive heart failure, liver failure, hypoalbuminemia, nephrotic syndrome, malignancy, infection, and autoimmune disorders.

A key factor in evaluation of non-CSF pleural effusions is determining whether it is transudative versus exudative, narrowing the differential diagnosis (Villena Garrido et al. 2014). However, with CSF effusion, the fluid may be either transudative or exudative (Chuen-im et al. 2012). CSF effusion is often yellow-tinged and can be confirmed by the presence of beta-2 transferrin, one of the most abundant polypeptides in human CSF, although false negative results have been reported (Taub and Lavyne 1994).

Pleural effusions secondary to CSF extravasation in the setting of VP shunts can occur by one of three mechanisms: (1) intrathoracic trauma during shunt placement; (2) migration of the peritoneal catheter into the thoracic cavity; (3) pleural effusion secondary to increased intraabdominal pressure due to CSF ascites (Taub and Lavyne 1994). Ours is the 38th reported case of CSF pleural effusion (Obrador and Villarejo 1977; Cooper 1978; Agha et al. 1983; Lourie and Bajwa 1985; Anegawa et al. 1986; Gaudio et al. 1988; Dickman et al. 1989; Savolaine and Khimji 1991; Meeker and Barnett 1994; Gupta and Berry 1994; Doh et al. 1995; Johnson and Maxwell 1995; Martin et al. 1997; Mayoralas Alises et al. 1999; Di Roio and Mottolese 2000; Akyuz et al. 2004; Samdani et al. 2005; Rahimi Rad et al. 2007; Kim et al. 2008; Ergun et al. 2008; Kiran et al. 2010; Glatstein et al. 2012; Globl 1978; Ceccotti et al. 1981; Trappe et al. 1988; Faillace and Garrison 1998; Hadzikaric et al. 2002; Muramatsu and Koike 2004; Adeolu et al. 2006; Born et al. 2008; Smith and Cohen 2009; Kocaogullar et al. 2011; Patel et al. 2011; Chuen-im et al. 2012; Ulus et al. 2012; Sekiguchi et al. 2013). A majority of these cases (58 %) were due to peritoneal catheter migration into the thorax. Nine (24 %) of these cases had associated ascites. The remainder (18 %) is assumed to be secondary to intrathoracic trauma at the time of shunt placement (Table 1).

**Table 1:** Summary of literature

	Study	Etiology of effusion
1	Obrador et al.	Migration of catheter
2	Cooper et al.	Migration of catheter
3	Agha et al.	Migration of catheter
4	Lourie et al.	Migration of catheter
5	Anegawa et al.	Migration of catheter
6	Gaudio et al.	Migration of catheter
7	Dickman et al.	Migration of catheter
8	Savolaine et al.	Migration of catheter
9	Meeker et al.	Migration of catheter
10	Gupta et al.	Migration of catheter
11	Doh et al.	Migration of catheter
12	Johnson et al.	Migration of catheter
13	Martin et al.	Migration of catheter
14	Mayorales et al.	Migration of catheter
15	Di Roio et al.	Migration of catheter
16	Akyuz et al.	Migration of catheter
17	Samdani et al.	Migration of catheter
18	Rahimi Rad et al.	Migration of catheter
19	Kim et al.	Migration of catheter
20	Ergun et al.	Migration of catheter
21	Kiran et al.	Migration of catheter
22	Glatstein et al.	Migration of catheter
23	Globl et al.	Abdominal ascites
24	Ceccotti et al.	Abdominal ascites
25	Trappe et al.	Abdominal ascites
26	Faillace et al.	Unclear
27	Hadzikaric et al.	Unclear
28	Muramatsu et al. (patient 1)	Abdominal ascites
29	Muramatsu et al. (patient 2)	Abdominal ascities
30	Adeolu et al.	Unclear
31	Born et al.	Abdominal ascites
32	Smith et al.	Abdominal ascites
33	Kocaogullar et al.	Unclear
34	Patel et al.	Unclear
35	Cheun-im et al.	Unclear
36	Ulus et al.	Unclear
37	Sekiguchi et al.	Abdominal ascities
38	Henningfeld et al. (this study)	Abdominal ascites

Development of pleural effusions due to the first two mechanisms is intuitive. The third mechanism, however, requires additional pathophysiology, including ineffective peritoneal absorption of CSF, a communication from the abdominal cavity, and ineffective pleural absorption of fluid. As ascites accumulate, abdominal hydrostatic pressure increases, eventually exceeding intrathoracic pressure, forcing CSF into the thorax through a diaphragmatic defect. Pleural effusions persist if pleural absorption is inadequate.

Diaphragmatic defects include the hernia of Bochdalek, the hernia of Morgagni, central diaphragmatic hernia, diaphragmatic eventration, and diaphragmatic agenesis. The former two are the most common and represent over 80 % of congenital diaphragmatic defects (Keijzer and Puri 2010; Schumacher and Gilbert 2009). In addition to these there are acquired diaphragmatic defects which may be secondary to previous diaphragm intervention, chest tube placement, pacemaker implantation, or VP shunt placement (Blitz and Louie 2009). In our case both chest tube placement and pacemaker implantation may have led to an acquired diaphragmatic defect.

## Conclusion

We present a case of right sided pleural effusion due to CSF extravasation from the abdomen via a diaphragmatic defect in an infant with a VP shunt. Additionally, the child had TOF requiring neonatal repair, necessitating consideration of right sided cardiac hemodynamics before conversion from VP to a VA shunt. This case highlights the need to consider CSF extravasation as a cause of pleural effusion in children with VP shunts and demonstrates that VA shunts appear to be safe and effective in children with CHD and otherwise normal right ventricular function. Long-term follow up should include particular attention to the development of pulmonary hypertension which may result from thromboembolic events related to the VA shunt.

## Abbreviations

ECMO: extracorporeal membrane oxygenation
VP: ventriculoperitoneal shunt
CSF: cerebrospinal fluid
VA: ventriculoatrial
TOF: tetralogy of Fallot
IVH: intraventricular hemorrhage
CHD: congenital heart disease
PICU: pediatric intensive care unit
CVP: central venous pressure
